# Effect of Heat Input on Microstructural Evolution and Impact Toughness of the Simulated CGHAZ for a Novel Q690 MPa V-N Medium and Heavy Plate

**DOI:** 10.3390/ma18051148

**Published:** 2025-03-04

**Authors:** Yang Liu, Heng Ma, Zhaoyu Wang, Xuehui Chen, Xiaoxin Huo, Hongyan Wu, Linxiu Du

**Affiliations:** 1 The State Key Laboratory of Rolling and Automation, Northeastern University, Shenyang 110819, China; 2010144@stu.neu.edu.cn (Y.L.); wangzhaoyu5603@163.com (Z.W.); wuhy@ral.neu.edu.cn (H.W.); 2 Laiwu Iron and Steel Group, Yinshan Steel Co., Ltd., Jinan 271104, China; maheng418@126.com (H.M.); oaks2000@sohu.com (X.H.); 3 Central Iro and Steel Research Institute Co., Ltd., Beijing 100081, China; chenxuehui@vip.sina.com

**Keywords:** V-N microalloyed steel, impact property, M/A constituents, Q690 MPa medium and heavy plate, intragranular acicular ferrite

## Abstract

In order to find the optimal heat input for simulating the welding of the coarse-grained heat-affected zone (CGHAZ) of a novel Q690 MPa V-N microalloyed medium and heavy plate, the study investigated the precipitation of V (C, N), microstructural changes, and impact toughness under five different heat inputs (E). The results show that in the CGHAZ, as the heat input increases, the dominant microstructure changes from intragranular acicular ferrite (IGAF) and lath bainitic ferrite (LBF) to polygonal ferrite (PF) and a small amount of IGAF. At the same time, the area fraction of the brittle phase martensite/austenite (M/A) constituents increased from 4.96% to 7.95% as heat input increased, and the microhardness difference between the M/A constituents and the matrix significantly increased. In addition, with the E increases, the fraction of high-angle grain boundaries (HAGBs), which can hinder crack propagation, increases from 59.2% to 62.2% and then decreases from 62.2% to 49.3%. Moreover, the impact toughness of the simulated CGHAZ of the Q690 MPa V-N microalloyed medium and heavy plate first increases from 62 J to 100 J and then decrease to 20 J.

## 1. Introduction

High-strength low alloy medium and heavy plates (HSLA) have been widely used in engineering machinery, structural construction, wind power generation, and heavy-section oil pipelines due to their appropriate strength and toughness [1,2]. Therefore, high-grade medium and heavy plates, especially those with a strength of 690 MPa, received increasing attention and application. Researchers are working on steels that have an exceptional combination of strength and toughness with improved service performance by reducing the yield ratio (yield strength/ultimate tensile strength) [3]. The combination of high strength and high toughness is jeopardized during the welding thermal cycle process because the heat-affected zone (HAZ), which is created by fast heating and prolonged exposure to high temperatures, shows decreased toughness. The studies found that the coarse-grained heat-affected zone (CGHAZ) close to the weld melt line experiences significant grain growth due to peak temperatures as high as 1350 °C or higher, which is therefore considered the region with the poorest toughness [4]. In particular, CGHAZ contains brittle and coarse microstructures such granular bainitic ferrite (GBF), Widmanstatten ferrite, and high-hard martensite/austenite (M/A) elements because of the high temperatures and comparatively high Si and Cr levels [5]. Qi et al. [6] showed that the formation of the second phase had an important influence on the microstructure evolution of the heat affected zone of Q690 steel plate. It is generally believed that these brittle phases (M/A and larger precipitates) of the microstructure are normally positively correlated with heat input (*E*) [7,8]. For C-Mn steel, a common method is to introduce fine, dispersed nonmetallic inclusions or precipitates, such as Ti, Ce, and V carbides, sulfides, or nitrides in the CGHAZ region to restrict prior austenite grains (PAGs) or/and promote intragranular acicular ferrite (IGAF) phase transition, thereby enhancing the impact toughness of the steel plate [9,10]. However, these nonmetallic inclusions are added or formed during the melting process. Due to the complexity of industrial melting and casting processes, it is difficult to control them accurately.

The complex microstructure within IGAF can effectively deflect or hinder crack propagation, and at the same time refine the internal grain structure, thereby enhancing the impact toughness of the CGHAZ. The amount of IGAF is closely related to the toughness of the steel plate. The formation of IGAF is significantly influenced by PAGS, the quantity and type of inclusions, and the cooling rate. Shi et al. [1] found that by adding different amounts of N to low-carbon V microalloyed steel, VN formed on Ti (C, N) in the CGHAZ under different heat inputs, resulting in the composite precipitation of (Ti, V) (C, N), which provided IGAF with more nucleation sites and enhanced the mechanical properties of the steel plate. Research found that E (corresponding to the cooling rate or the t_8/5_ representing the time during the cooling from 800 to 500 °C, of the welding thermal cycle) greatly influenced the precipitation potential of V (C, N) in CGHAZ and its ability to promote the IGAF phase transformation [11]. Hu et al. [12] reported that adding 0.1 V and 0.018 N to low-carbon steel, with t_8/5_ ranging from 10 s to 120 s, resulted in significant nucleation of V (C, N) for IGAF when t_8/5_ was 20 s, and the main microstructure was IGAF, which can effectively stop crack propagation and enhance impact toughness. Wang et al. [13] found that when X80 pipeline steel was added 0.14 V and 0.017 N, the t_8/5_ ranged from 20 s to 327 s, and the CGHAZ obtained the best impact toughness performance due to its microstructure, which consists of 85.79% IGAF and a trace amount of brittle phase M/A constituents when t_8/5_ was 20 s.

The applications of V-N microalloyed medium and heavy plates with low yield ratio are more and more widely due to the good internal microstructure uniformity. Our previous research developed a new VN composition system combined with TMCP technology to produce the plate with the strength of 690 MPa, and analyzed its microstructure evolution and precipitation behavior, but did not conduct in-depth research on welding performance [14]. However, the precipitation of VN or V (C, N), the type and quantities of ferrite, as well as the shape of M/A constituents in the CGHAZ change with variations in heat input during the welding process. At the same time, the mechanisms of these precipitates and microstructure changes affecting the variation in impact properties are rarely clear-cut and require careful research. A series of welding thermal cycle simulations at various E will be performed on a medium and heavy plate with a low yield ratio, a strength of 690 MPa, 0.1 weight percent (wt. %) V, and 0.01 wt. N. The connection between precipitation, microstructure, and impact properties in the CGHAZ was studied in depth. This provides a novel Q690 MPa V-N medium and heavy plate with improved weldability.

## 2. Experimental Procedures

### 2.1. Material

Table 1 displays the V-N experimental steel’s nominal chemical composition in weight percentage. Especially in order to encourage the varied nucleation of acicular ferrite on nanometer V (C, N) nanoparticles in CGHAZ, 0.1%V and 0.01%N were added to the experimental steel [15,16]. Equations (1) and (2) were used to determine the welding crack susceptibility index (Pcm) and the equivalent carbon content (Ceq), respectively [17].

(1) $$C_{\mathrm{eq}}\;=\;C+\frac{\mathrm{Mn}}{6}+\frac{\mathrm{Cr}+\mathrm{Mo}+V}{5}+\frac{\mathrm{Ni}+\mathrm{Cu}}{15}$$

(2) $$P_{\mathrm{cm}}\;=\;C+\frac{\mathrm{Si}}{30}+\frac{\mathrm{Mn}+\mathrm{Cu}+\mathrm{Cr}}{20}+\frac{\mathrm{Ni}}{60}+\frac{\mathrm{Mo}}{15}+\frac{V}{10}+5B$$

After being melted in a vacuum induction furnace, the steel was formed into 255 kg ingots. The billet was subjected to two phases of controlled rolling. The first step involved five passes through Shandong Steel Company’s 4500 mm rolling machine to roll the billet to an intermediate thickness of 150 mm. The intermediate billet was rolled in eight passes to 30 mm-thick sheets in the second step after air cooling to 820–870 °C. The final temperature was maintained between 805 and 820 °C. Subsequently, the steel was subjected to two-stage continuous cooling. In the first stage, the slat was water-cooled at a rate of 20 °C/s to 490 °C [14]. The slat was cooled to room temperature in the second stage at a rate of 0.3 °C per second.

### 2.2. Welding Simulation Procedure

Samples measuring 11 × 11 × 55 mm were cut from the quarter of the V-N experimental steel plate in a rolling direction for the welding heat cycle simulation. An MMS-300 thermomechanical simulator was used to generate welding thermal simulation tests with various E (the MMS-300 is a self-developed equipment of RAL state key laboratory of Northeastern University, its control temperature is accurate to ±1 °C and its performance is the same as that of classical welding equipment. RAL, Shenyang, China). The 2-dimensional Rykalin mathematical model was used to compute the welding thermal cycle curves in order to represent the welding of plates that were 15 mm thick [12]. Considering the connection between E and t_8/5_ shown in Equation (3), the corresponding various heat inputs were 15.7 kJ/cm, 20.2 kJ/cm, 28.6 kJ/cm, 49.5 kJ/cm, and 70.1 kJ/cm, respectively [12,18].

(3) $$E\;=\;d\sqrt{\frac{4\pi l\rho ct_{8/5}}{(1/{\left(500-T_{0}\right)}^{2})-(1/{\left(800-T_{0}\right)}^{2})}}$$

**Figure 1:**
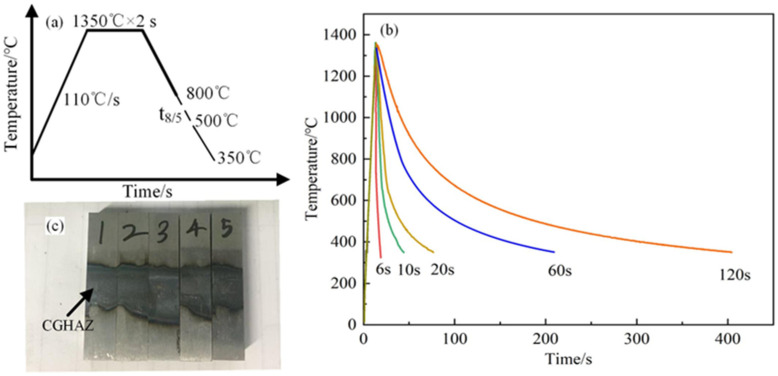
Thermal cycle curves for welding in simulated CGHAZ. (**a**) Schematic diagram of welding simulation process. (**b**) The actual welding curve. (**c**) CGHAZ of Charpy impact specimen.

Here, E is the welding heat input (J/cm); *l* is the thermal conductivity of the steel, which was determined to be 0.5 W/(cm·°C), *ρ* stands for density, and 7.8 g/cm^3^ was selected, and c stands for steel’s specific heat capacity, which was determined to be 1 J/(g·°C) [12]. The pre-heating temperature of 20 °C was chosen, as shown by $T_{0}$, and d denotes the plate thickness, which was chosen as 1.5 cm. During the test, the heat input is controlled by controlling the value of t_8/5_. The experimental data obtained by using Equation (3) have an important guiding role for the actual welding process. Therefore, the t_8/5_ was selected as 6 s, 10 s, 20 s, 60 s, and 120 s (Table 2) [17]. Figure 1a is the schematic diagram of the welding experiment simulation process. The samples were heated to 1350 °C at the rate of 110 °C/s. Furthermore, the specimens were held for 2 s at 1350 °C. A type-K thermocouple wire that was percussion welded in the middle was used to regulate the temperature, and the final temperature was 350 °C. The detailed welding thermal cycles are shown in Figure 1b. In order to ensure the accuracy, five tests were carried out for each group of parameters, three of which were tested for mechanical properties.

### 2.3. Microstructural Characterization and Mechanical Properties

The samples for microstructural studies were examined by a Zeiss Ultra 55 scanning electron microscopy (SEM, Carl Zeiss, Oberkochen, Germany) and a Leica DMIRM optical microscope (OM, Leica, Weztlar, Germany). The element distribution was observed by a JXA-8530F electron probe microanalyzer (EPMA, JEOL. Co., Ltd., Tokyo, Japan), which was operated at 20 kV, 2 × 10^−8^ A, and a step size of 50 nm. HKL-Channel 5 software was used to analyze the electron backscatter diffraction (EBSD) maps, which were created using Flamenco software (Channel5 2019 v5.12) at a 0.15 μm step size. The specimens for OM, SEM, and EPMA analyses were mechanically polished using the standard metallographic procedure and etched with a 4 vol% Nital solution for 15–20 s, while the specimens for EBSD analyses were mechanically polished, followed by electropolishing using an electrolyte of 87.5 vol% CH_3_CH_2_OH and 12.5 vol% HCLO_4_ at room temperature. The residual stress layers are removed in 18 s with a current of about 0.7 A. The Φ3 mm diameter is ground to a 40 μm thickness, and the twin jet is polished by 12.5 vol% perchloric acid alcohol solution at −20 °C and 27 V. The transmission electron microscopy (TEM) samples are observed by using an FEI Tecnai G2 F20 (FEI, Hillsboro, USA) microscope at an accelerating voltage of 200 kV.

Charpy V-notch impact tests were conducted at −20 °C using standard specimens (dimensions: 10 × 10 × 55 mm^3^) on an ASTM E23 pendulum impact tester (INSTRON, Boston, MA, USA) [19]. The opening position of Charpy impact sample is in CGHAZ, as shown in the Figure 1c. Using Image-Pro software, (Image-Pro Plus 6.0) over 1000 M/A constituents across 20 fields of sight were counted, and the changing trend of the average size and area proportion of M/A constituents in the CGHAZ was measured.

## 3. Results

### 3.1. Effect of Heat Input on Microstructural Evolution and Precipitation

**Figure 2:**
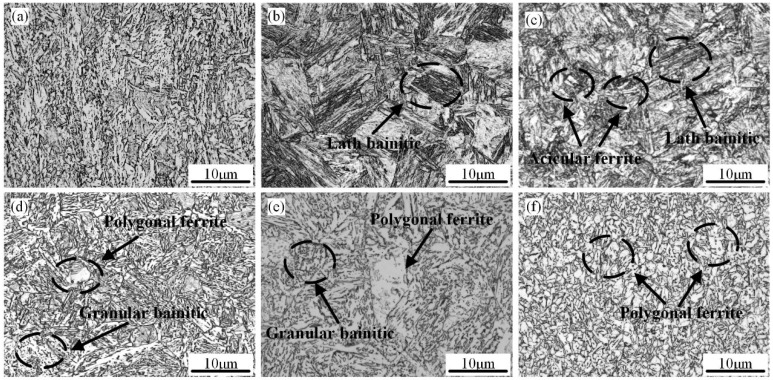
OM micrographs of simulated CGHAZ samples at various t_8/5_ and as-processed V-N heavy plate (**a**) as-processed V-N heavy plate; (**b**) 6 s; (**c**) 10 s; (**d**) 20 s; (**e**) 60 s; and (**f**) 120 s.

**Figure 3:**
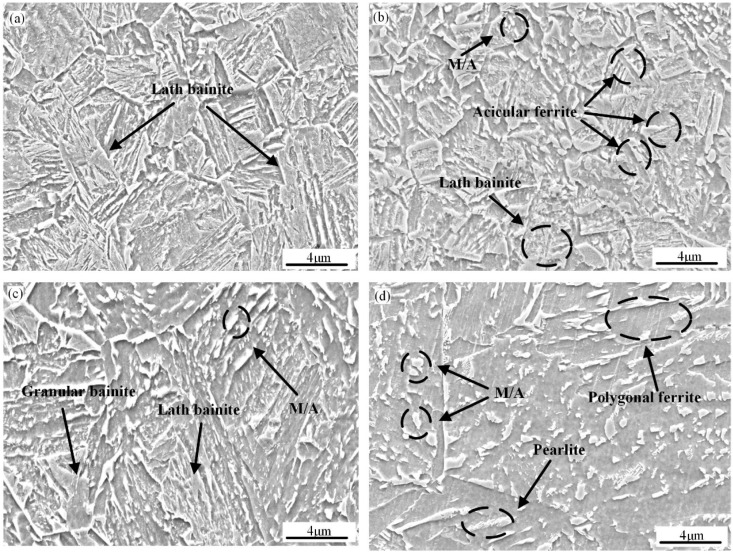
SEM micrographs of simulated CGHAZ samples at various t_8/5_: (**a**) 6 s; (**b**) 10 s; (**c**) 20 s; and (**d**) 120 s.

Figure 2 and Figure 3 show the OM and SEM-dominant microstructure of the simulated samples at various t_8/5_, respectively. Acicular ferrite, lath bainite, granular bainite, and a few martensite/austenite components (M/A) constitute the majority of the base metal’s microstructure in the welding thermal simulation sample (Figure 2a). However, when t_8/5_ is 6 s, the welded specimen’s CGHAZ primarily consists of lath bainite, with traces of M/A components visible (Figure 2b and Figure 3a). The microstructure is composed of fine intragranular acicular ferrite (IGAF), lath bainite ferrite (LBF), and a little granular bainite ferrite (GBF) when the t_8/5_ is 10 s (Figure 2c). At the same time, the amount of M/A constituents rose significantly and is mainly in the stripes (Figure 3b). Lath bainite ferrite is composed of fine bainitic lath and grows from prior austenite boundaries. When the t_8/5_ is 20 s, the microstructure is composed of GBF, LBF, some small size polygonal ferrite (PF), and some massive M/A constituents (Figure 2d and Figure 3c). When t_8/5_ reaches 60 s, PF, GBF, and blocky M/A constituents are the main metallographic microstructure of the simulated CGHAZ (Figure 2e). When the t_8/5_ increases to 120 s, PF, IGAF, pearlite (P), and massive M/A constituents are mostly the microstructure of the sample (Figure 2f). As the t_8/5_ increases, the number of M/A constituents monotonically increases (Figure 3d).

**Figure 4:**
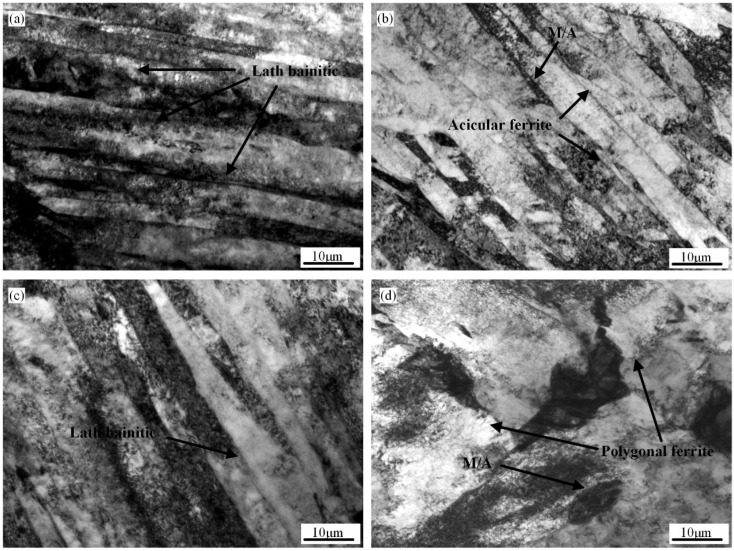
The TEM micrographs of microstructure at various t_8/5_: (**a**) 6 s; (**b**) 10 s; (**c**) 20 s; and (**d**) 120 s.

Figure 4 shows the TEM morphologies of the CGHAZ simulation at different t_8/5_s of 6 s, 10 s, 20 s, and 120 s. When t_8/5_ is 6 s, the main lath bainite has the same orientation (Figure 4a). When the t_8/5_ is 10 s and the IGAF width is 80~150 nm, Figure 4b shows that the IGAF contains high-density dislocations. At a t_8/5_ equal to 20 s, the width of the IGAF grows obvious (Figure 4c). When the t_8/5_ increases to 120 s, PF becomes mostly the microstructure of the CGHAZ sample (Figure 4d). As t_8/5_ rises, the size of PF significantly increases. The size is greatly increased and the M/A constituents shift from lath to block as the t_8/5_ grows from 6 s to 120 s. Furthermore, toughness is negatively impacted by the M/A constituents.

**Figure 5:**
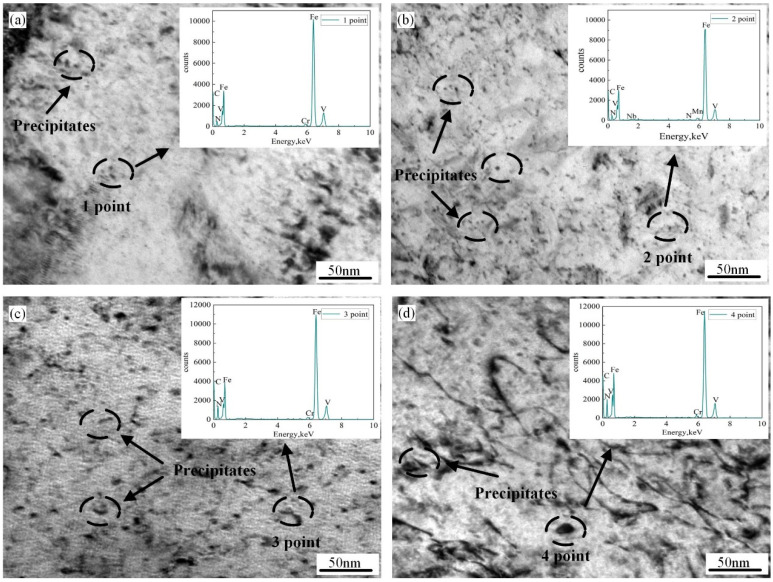
The TEM micrographs of precipitates in the simulated CGHAZ samples at different t_8/5_: (**a**) 6 s; (**b**) 10 s; (**c**) 20 s; and (**d**) 120 s.

Zhang et al. found that V (C, N) precipitates disintegrate at 1230 °C by employing the Thermo-Calc software (TCFE V9.3 and MOBFE4 v4.0) [20]. The welding thermal simulation sample will first be heated to 1350 °C, at which time the V (C, N) precipitate in the structure has been completely dissolved. The TEM micrographs of precipitates in the simulated samples at various are shown in Figure 5. When the t_8/5_ is 6 s, there are a tiny quantity of precipitation dispersed in the simulated CGHAZ and the 1 point precipitate is V (C, N) by EDX (Figure 5a). According to Figure 5b, a large number of precipitates form when t_8/5_ is 10 s. The size of the precipitates is 3–10 nm, and the 2-point precipitate is V (C, N) by EDX. When the t_8/5_ is 20 s, the size of the precipitates is 20–30 nm, and the 3-point precipitate is V (C, N) by EDX (Figure 5c). When the t_8/5_ reaches 120 s, the amount and size of precipitates increase significantly (Figure 5d). When t_8/5_ is 120 s, the size of the precipitates is 40–50 nm. Moreover, the 4-point precipitates are V (C, N) by EDX. The lower temperature range of α and (α + γ) was where these tiny precipitates were primarily generated. By interacting with the dislocations and preventing their mobility, the nanoscale V (C, N) precipitates that form in the matrix and on the dislocations would strengthen steel.

**Figure 6:**
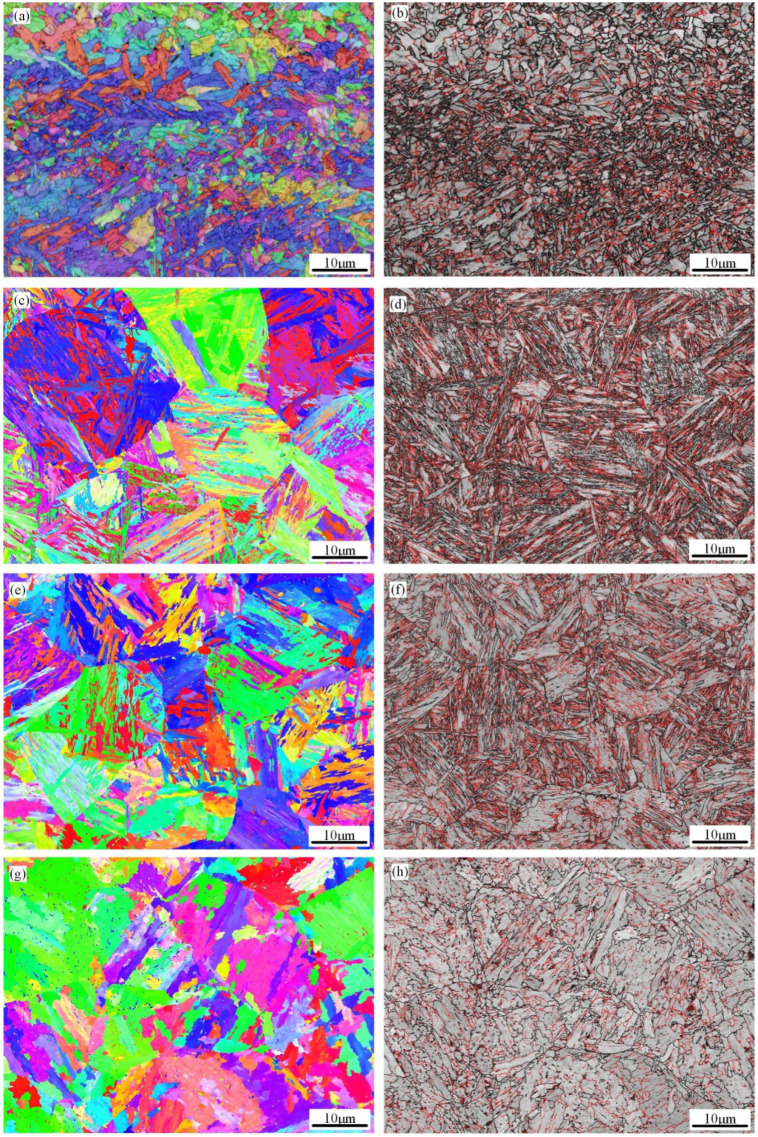
EBSD analyzes of the simulated CGHAZ at various t8/5: (**a**,**b**) 6 s; (**c**,**d**) 10 s; (**e**,**f**) 20 s; and (**g**,**h**) 120 s. red color represents the crystal plane index of (0 0 1); blue color represents the crystal plane index of (1 1 1); green color represents the crystal plane index of (1 0 1).

**Figure 7:**
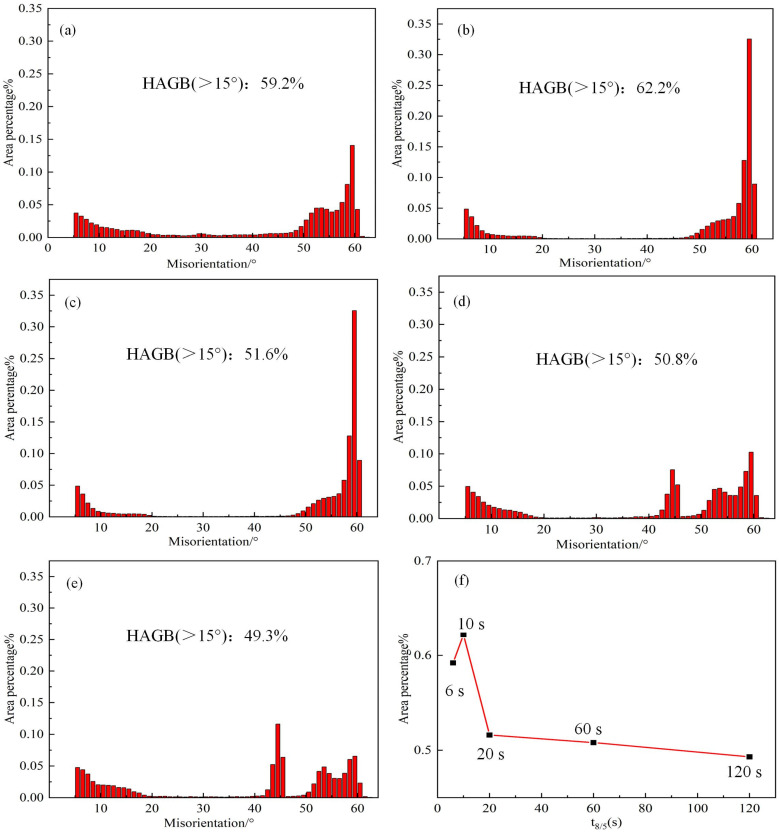
The high misorientation angle vs. percentage of simulated CGHAZ at various t_8/5_ analyzed by EBSD: (**a**) 6 s; (**b**) 10 s; (**c**) 20 s; (**d**) 60 s; (**e**) 120 s; and (**f**) the trend chart of high misorientation angle and t_8/5_.

To determine the microstructural characteristics of the simulated CGHAZ at various t_8/5_s, EBSD crystallographic investigations are conducted. Figure 6 displays high-angle grain boundary (HAGB) and inverse pole figure (IPF) maps, whereas Figure 7 displays the equivalent graphs of the misorientation angle versus percentage. In the orientation image quality map, different colors represent different crystallographic orientations and are accordingly identified by grain boundary misorientation. Herein, the bold black line represents high grain boundaries of 15° and greater than 15° (high-angle grain boundaries, HAGBs), and the red line represents the low grain boundaries of 2–15° (low-angle grain boundaries, LAGBs). The simulated CGHAZ samples have a lath bainite and IGAF microstructure when the t_8/5_ is 6 s. However, because of the fast rate of cold, the IGAF has little time to grow and is small (Figure 6a,b). The microstructures of the simulated CGHAZ samples are primarily made up of intragranular acicular ferrite with various orientations when t_8/5_ is 10 s, as shown in Figure 6c,d. Moreover, these microstructures with different orientations can effectively lead to the deflection direction of crack propagation to improve the impact toughness. Figure 6e,f shows that when t_8/5_ is 20 s, the PF begins to appear in the microstructure of the simulated sample. When the t_8/5_ increases to 120 s, the microstructure of the simulated CGHAZ samples is primarily made up of PF, IGAF, and P (Figure 6g,h). While minor misorientation boundaries cannot cause a discernible deviation of the cleavage cracks, a grain packet with high orientation can successfully deflect or even stop the spread of cleavage microcracks [21,22]. Figure 7 shows the number fraction of HAGBs following the sequence (t_8/5_ = 10 s) > (t_8/5_ = 6 s) > (t_8/5_ = 20 s) > (t_8/5_ = 60 s) > (t_8/5_ = 120 s).

### 3.2. Mechanical Properties

Table 3 displays the simulated CGHAZ’s total CVN impact energy as a result of t_8/5_, ranging from 6 to 120 s. The sample’s CVN impact energy is 100 J when t_8/5_ is 10 s. In addition, the impact energy is 62 J, 50 J, 30 J, and 20 J, respectively, subjected to t_8/5_ 6 s, 20 s, 60 s, and 120 s. The mean CVN impact energy value drops from 100 J to 20 J as t_8/5_ increases from 10 s to 120 s; the same trend in impact toughness was also observed in earlier studies [12,23].

**Figure 8:**
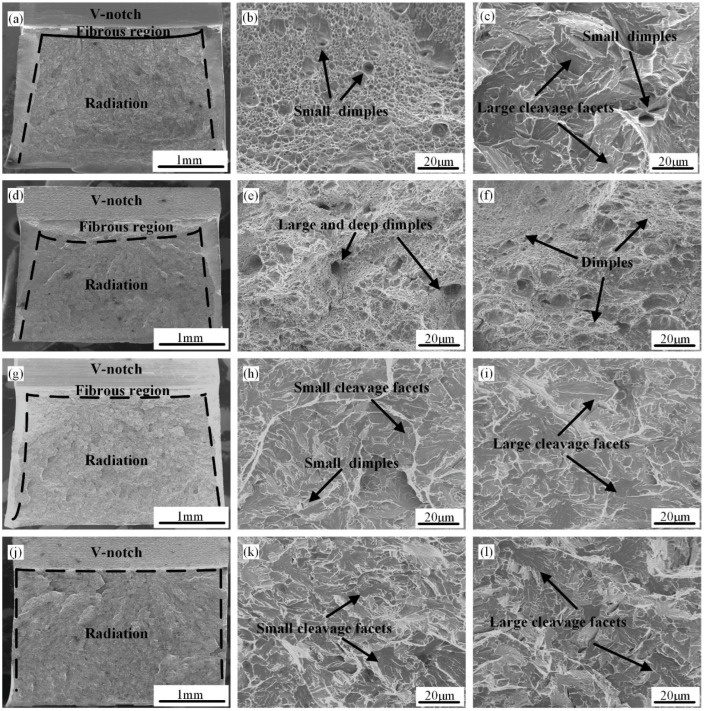
The simulated CGHAZ’s microfracture and microfracture morphologies in the V-N experimental steel at various t_8/5_s: (**a**,**d**,**g**,**j**) low-magnification picture; (**b**,**e**,**h**,**k**) high-magnification image of fiber area; (**c**,**f**,**i**,**l**) high-magnification image of the radiation; (**a**–**c**) fracture surface at t_8/5_ of 6 s; (**d**–**f**) fracture surface at t_8/5_ of 10 s; (**g**–**i**) fracture surface at t_8/5_ of 20 s; and (**j**–**l**) fracture surface at t_8/5_ of 120 s.

The simulated CGHAZ’s microfracture and microfracture morphologies in the V-N experimental steel at t_8/5_ of 6 s, 10 s, 20 s, and 120 s are displayed in Figure 8. The macro morphologies of the impact are displayed in Figure 8a,d,g,j, the fibrous region and radiation region are distinguished by black lines, and the impact fractures at the 6 s,10 s, and 20 s conditions all have a fibrous region and radiation region. Moreover, when the t_8/5_ increases to 120 s, the impact fractures zone is still composed of a radiation region and fibrous region, while the radiation zone is extremely small, and almost all of them are in the radiation zone. It is evident from the impact fracture that the toughness zone is clearly greater when t_8/5_ is 10 s. This is in line with what the impact test findings show. Additionally, the radiation and fibrous zones are observed under severe magnification. The standard microscopic fractographies of brittle and ductile zones are displayed in Figure 8d–f. When t_8/5_ is 6 s, the fibrous regions consist of some small dimples, and the radiation regions include some shallow dimples and cleavage facets (Figure 8b,c). When t_8/5_ is 10 s, the fibrous region and radiation region both consist of large and deep dimples (Figure 8e,f). It can absorb energy and increase toughness under pressure because the radiation zone’s cleavage facets are very tiny and connected by tear ridges and dimples. When t_8/5_ is 20 s, the fibrous regions include some tiny, shallow dimples, whereas the radiation regions consist of small tera ridges and large river-patterned cleavage facets (Figure 8h,i). At t_8/5_ of 120 s, the fibrous region and radiation region are basically composed of small cleavage facets and large cleavage facets, and there are almost no dimples, which is one of the reasons for the sharp decline of impact energy. In general, the samples with t_8/5_ = 10 s have deeper, more uniform dimples and cleavage facets that are much finer than those with t_8/5_ = 6 s, t_8/5_ = 20 s, t_8/5_ = 60 s, and t_8/5_ = 120 s. At the same time, with the increase in t_8/5_, the number of fiber zones and dimples that can absorb impact energy in the fracture of the simulated welding heat-affected zone first increased and then gradually declined, and the impact toughness of experimental steel also showed the same rule. Above all, the best impact toughness is obtained when t_8/5_ is 10 s.

## 4. Discussion

### 4.1. Effect of Heat Input on the Microstructure

**Figure 9:**
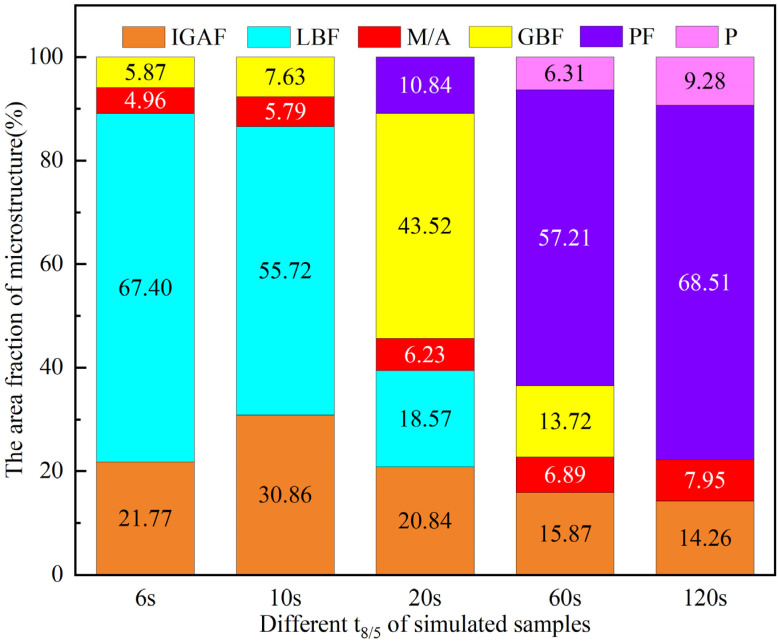
Each microstructure’s area percentage in CGHAZ with various t_8/5_s.

More than 30 microstructure diagrams are examined and computed using Image-Pro software to examine the impact of varying heat input on the size and proportion of the microstructure of welding thermal simulation samples. The pertinent statistical findings are displayed in Figure 9. When the t_8/5_ is 6 s, the main structure of the samples is 67.40% LBF, 21.77% IGAF, and 5.87% GBF. When the t_8/5_ is 10 s, the proportion of LBF and IGAF in CGAZ reach 55.72% and 30.86%, respectively. However, these two values are 18.57% and 20.84% with the increase in t_8/5_ to 20 s, and 43.52% LBF microstructure became the main structure of the samples, and PF microstructure began to appear. Bainite ferrites tend to preferentially nucleate at positions with irregular atomic arrangement and lattice distortion in PAGBs, but the γ → GBF is transformed at a higher temperature than the γ → LBF [24]. At the t_8/5_ of 10 s, there is a lower transition and greater degree of supercooling, so the proportion of the LBF of the CGHAZ is significantly higher than when the t_8/5_ is 20 s. A substantial number of GBF is formed in the microstructure when t_8/5_ is 20 s as a result of the degree of supercooling decreasing as the phase transition temperature rises. Furthermore, the diffusion of carbon atoms is facilitated by the prolonged residence time in the high temperature range at 60 and 120 s, and the PF transition at PAGBs is encouraged by the relatively low degree of super-cooling. Therefore, the proportion of PF reached 57.21% and 68.51% at t_8/5_ of 60 s and 120 s, respectively. At different t_8/5_s, IGAF microstructures were observed in the simulated welding samples, while the proportion of IGAF varied among different t_8/5_s. This is because the experimental steel’s composition was enhanced with V and N, and the IGAF phase transition may be promoted by the V (C, N) precipitation that produces V-microalloyed steel plates [25]. Previous research confirmed the V (C, N) precipitate is an MC-type carbide with a crystal structure FCC-NaCl type. Additionally, the V (C, N) precipitate and the ferrite matric is the cube-on-cube orientation relationship: [001]_α_//[1$\overset{-}{1}$0]_V(C,N)_, which can effectively reduce the interfacial structural energy for the nucleation of IGAF [17,26]. In addition, there is a 3.4% lattice matching between VN and α-Fe, which is less than the 6% lattice matching that can effectively promote nucleation [27,28]. Additionally, the content of C atoms surrounding the matrix is consumed by the production of V (C, N) precipitates, decreasing austenite stability and raising the chemical driving force for heterogeneous IGAF nucleation [29]. In addition, it has been confirmed through Figure 5 that a large amount of V (C, N) particles exists in the samples of various t_8/5_s, and therefore, each sample contains an IGAF microstructure. Due to the effect of V (C, N) particles, many IGAFs are preferentially nucleated when t_8/5_ is 10 s. As t_8/5_ increases to 120 s, the precipitation size increases and the nucleation ability is enhanced. However, due to the long residence time at high temperatures, a large amount of PF is formed at PAGs, and then a small amount of IGAF is formed at a lower transformation temperature. The formation of IGAF not only requires nucleation sites, but also appropriate supercooling. When t_8/5_ is 120 s, the cooling rate is greater, so the proportion of IGAF is significantly less than when t_8/5_ is 10 s, which agrees with the conclusions of Hu et al. [12].

**Figure 10:**
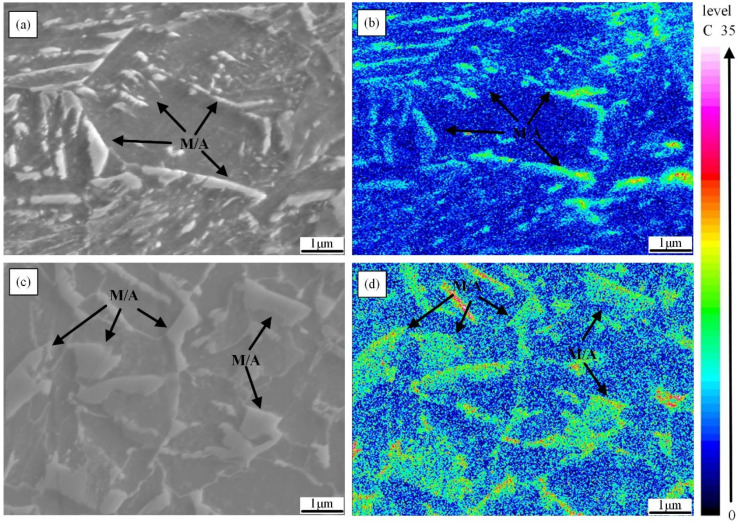
The carbon distributions and micrographs of EPMA maps for t_8/5_ of 10 s and 120 s: (**a**,**c**) 10 s; (**b**,**d**) 120 s.

The phase transformation from γ to α and $\gamma^{\prime }$ (retained austenite) is often accompanied by the diffusion of carbon atoms; because the solubility of carbon in γ phase is much greater than that in α phase, carbon atoms will diffuse into $\gamma^{\prime }$ during the phase transition process, resulting in the obvious enrichment of carbon atoms in $\gamma^{\prime }$ during the subsequent fast cooling process and becoming M/A constituents [30]. The amount and shape of M/A constituents have a significant impact on the impact toughness of CGHAZ because they are highly hard and frequently serve as crack initiation sites [31]. When t_8/5_ is 10 s and 120 s, the M/A constituents in the sample are displayed in Figure 10a,c. Because the M/A constituents are carbon-rich, electron probe microanalysis (EPMA) was performed (Figure 10b,d). In order to determine the proportion and effective diameter of M/A constituents, over 1000 M/A constituents in 20 ranges of view at various t_8/5_s were measured; the pertinent statistical findings are displayed in Figure 9 and Table 4. When t_8/5_ is 10 s, the diffusion of carbon atoms to $\gamma^{\prime }$ is through LBF, because the lath boundaries are recognized as a channel conducive to the diffusion of C. The diffusion rate of carbon along the borders is substantially higher than elsewhere throughout the fast cooling process, and eventually takes on a thin shape (Figure 10a) [32]. In addition, when t_8/5_ is 10 s, the proportion of preferentially formed IGAF is higher, which can divide $\gamma^{\prime }$ into smaller segments [33]. Furthermore, decreasing IGAF improves *f*_M/A_, but refines *d*_M/A_. Therefore, the diameter of M/A constituents is 2.26 μm, but the proportion of M/A constituents is 5.79% when t_8/5_ is 10 s. With the increase in the t_8/5_ to 120 s, microstructures such as GBF, PF, and P increase, becoming the main microstructure of the simulated samples, and therefore, the diffusion channels of carbon atoms decrease. The carbon atoms’ diffusion temperature and time both rise as the supercooling reduces, causing sufficient diffusion that forms the C-rich “γ” phase before changing into the square M/A constituents during the cooling process (Figure 10c). Therefore, the diameter of M/A constituents reduced to 1.75 μm, but the percentage of M/A constituents raised to 7.95% at t_8/5_ of 120 s.

### 4.2. Effect of Welding Heat Input on Impact Toughness

Generally speaking, the size and microstructure of precipitates have a major influence on changes in impact energy [34]. According to the study, impact energy rises as welding heat input increases from 6 to 10 s, but falls when welding heat input grows from 10 to 120 s. In addition, IGAFs comprising an interlocking structure of nonparallel laths and LBF in various orientations can successfully prevent cracks from spreading and hence increase impact resistance [35]. When t_8/5_ is 10 s, the V (C, N) precipitations formed by V and N in the experimental steels promote the phase transformation of IGAF at the appropriate cooling rate, so the impact energy of the CGHAZ is effectively increased. Nevertheless, the ability of GBF and PF to stop crack propagation and deflection is limited. As a result, as t_8/5_ changes from 10 s to 120 s, the microstructure changes from IGAF and LBF to GBF and PF, and the impact energy decreases.

**Figure 11:**
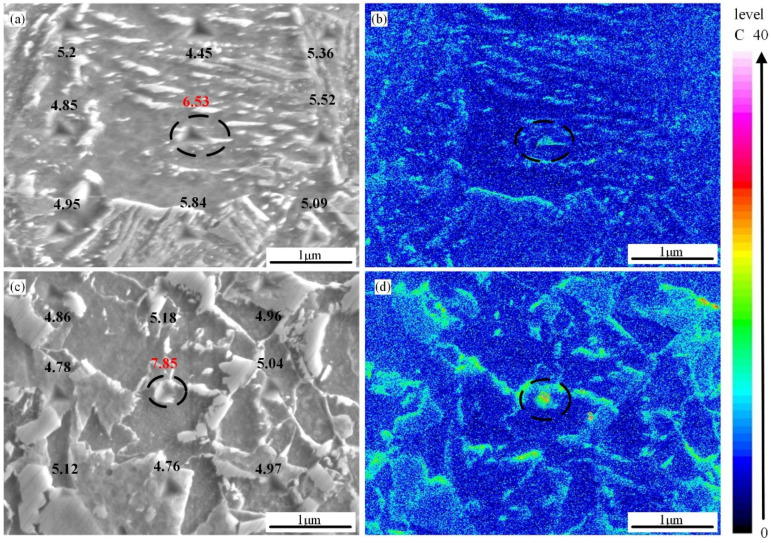
Nanoindentation micrograph and element distribution diagram under EPMA at various t_8/5_: (**a**,**b**) 10 s; (**c**,**d**) 120 s.

Both the soft and hard phases in the microstructure typically experience elastic deformation when the steel plates are loaded. However, the soft phase tends to produce plastic deformation more readily than the hard phase. As a result, stress concentrates at the interface between the soft and hard phases. With the continuous increase in the load, microcracks or/and microcleavages will form in these regions. Therefore, the hardness difference between the soft and hard phases in the microstructure has a significant impact on crack initiation [36]. In the study, with the increase in t_8/5_, the proportion of the hard-phase M/A constituents increases monotonously (Figure 9 and Table 4). The hard phase and soft phase in the microstructure under different t_8/5_s were studied by the nanoindentation test. Because the M/A constituents are carbon-rich, electron probe microanalysis (EPMA) was used to determine if the indentations were at the M/A constituents. Figure 11a,c shows the nanoindentations of the M/A constituents when t_8/5_ is 10 s and 120 s, and Figure 11b,d shows the corresponding EPMA maps. Table 5 shows the result of hardness. The hardness of the matrix structure diminishes as t_8/5_ increases, but the hardness of the M/A constituents increases. When t_8/5_ is 10 s, the variation in hardness between the matrix and M/A phase is 1.38 GPa. However, when t_8/5_ is increased to 120 s, the difference significantly increases to 2.89 GPa.

The grain boundary orientation encountered by a crack during its propagation plays a crucial role in the deflection or cessation of crack growth. High-angle grain boundaries (grain boundary angle > 15°) have been demonstrated in earlier research to efficiently absorb energy, changing the direction of crack propagation or preventing crack formation [37]. Based on the data acquired from EBSD, when t_8/5_ is 10 s, the number fraction of HAGBs is 62.2%, which is significantly higher than 49.3% when t_8/5_ is 120 s (Figure 7). The number of HAGBs increased with the increases in t_8/5_ from 6 s to 10 s, and decreased with the increase in t_8/5_ from 10 s to 120 s (Figure 7f). This is related to the proportion of IGAF in which IGAF boundaries are more than 15°. The proportion of IGAF at t_8/5_ = 10 s is significantly higher than that at t_8/5_ = 120 s. At the same time, the MED_MTA≥15°_ follows the sequence (t_8/5_ = 10 s) < (t_8/5_ = 6 s) < (t_8/5_ = 20 s) < (t_8/5_ = 60 s) < (t_8/5_ = 120 s) (Table 4). When t_8/5_ is 10 s, the MED_MTA≥15°_ is 5.07, which is significantly smaller than the value of 7.74 when t_8/5_ is 120 s. This indicates that, compared to t_8/5_ = 120 s, at t_8/5_ = 10 s, the length of MED_MTA ≥ 15°_ grain boundaries per unit area increases, leading to a significantly higher probability of the crack encountering HAGBs during propagation. This results in increased energy consumption, thereby enhancing the impact toughness of the simulated specimen. Moreover, cracks are more likely to propagate through the PF [17]. With the increase in t_8/5_, the proportion of PF structure increases, which is detrimental to impact toughness.

In conclusion, when t_8/5_ is increased from 6 s to 10 s, the proportion of IGAF in the simulated specimens gradually increases, and with the increase in t_8/5_ from 10 s to 120 s, the proportion of IGAF in the simulated specimens gradually increases, while the proportion of M/A increases, the diameter decreases, and the proportion of PF increases. These factors collectively lead to the best impact toughness of the simulated specimens when t_8/5_ is 10 s. In this study, the best welding process of the Q690 MPa V-N plate suitable for no-quenching and tempering was found. In addition, the production process can significantly reduce carbon emissions and save energy.

## 5. Conclusions

The changes in microstructure and impact characteristics of the CGHAZ were investigated as t_8/5_ increased from 6 s to 120 s for Q690 MPa V-N medium and heavy steel plates without quenching and tempering. The key findings of this study are summarized as follows:

(1) The V-N medium and heavy steel plates, without quenching and tempering, exhibit good weldability due to the addition of V and N, which form V (C, N) precipitates in the austenite region. These precipitates promote the formation of IGAF in the CGHAZ, enhancing the impact toughness of the steel plate.
(2) The microstructure changed from IGAF + LBF to GBF + IGAF and PF + IGAF when t_8/5_ increased from 6 s to 120 s. With the increase in t_8/5_, the content of IGAF in the microstructure first increases and then decreases, while the content of M/A constituents increases and the diameter decreases.
(3) With the increase in t_8/5_, the hardness difference between the M/A phase and the matrix increases, leading to a higher degree of micro-strain concentration, which provides favorable conditions for crack initiation and is detrimental to the toughness of the steel plate.
(4) When t_8/5_ is 10 s, the best impact energy of 100 J is obtained, which is a result of the combined effect of a higher proportion of IGAF and a smaller hardness difference between the hard phase and the matrix.

## Figures and Tables

**Table 1:** The chemical of V-N experimental steel (wt%).

C	Mn	Cr + Mo + Nb	V	Si	N	Ceq	Pcm
0.14	1.7	0.59	0.10	0.23	0.01	0.55	0.27

**Table 2:** Conversion between different heat input and t_8/5_.

t_8/5_ (s)	E (J/cm)
6	15.7
10	20.2
20	28.6
60	49.5
120	70.1

**Table 3:** CVN impact energy at different t_8/5_ of simulated samples (−20 °C).

t_8/5_ (s)	E (J/cm)	Impact Energy (J)
6	15.7	62 ± 2
10	20.2	100 ± 3
20	28.6	50 ± 4
60	49.5	30 ± 2
120	70.1	20 ± 3

**Table 4:** Quantifications results of different t_8/5_ of simulated samples.

t_8/5_/s	f_M/A_/%	d_M/A_/μm	f_MTA>15°_/%	MED_MTA≥15°_/μm
6 s	4.96 ± 0.18	2.84 ± 0.04	59.2	5.77
10 s	5.79 ± 0.20	2.26 ± 0.02	62.2	5.07
20 s	6.23 ± 0.30	2.03 ± 0.03	51.6	6.10
60 s	6.89 ± 0.15	1.84 ± 0.02	50.8	7.58
120 s	7.95 ± 020	1.75 ± 0.02	49.3	7.74

f_M/A_—the M/A constituents’ area portion, d_M/A_—average size of M/A constituents, MTA—the angle of misorientation tolerance, MED—the average equivalent diameter, f_MTA_ > 15°—the percentage of boundaries at MTA higher than 15°, and MEDMTA ≥ 15°—the average equivalent diameter of grains with boundaries at MTA higher than 15°.

**Table 5:** Summary of nanoindentation hardness of M/A constituent and matrix.

t_8/5_ (s)	M/A Constituents (GPa)	Matrix (GPa)	Difference (GPa)
10	6.53	5.15	1.38
120	7.85	4.96	2.89

## Data Availability

The data presented in this study are available on request from the corresponding author due to privacy.
